# Chloroplast Genome and Description of *Borodinellopsis insignis* sp. nov. (Chlamydomonadales, Chlorophyta), a Rare Aerial Alga from China

**DOI:** 10.3390/plants13223199

**Published:** 2024-11-14

**Authors:** Qiufeng Yan, Benwen Liu, Guoxiang Liu

**Affiliations:** 1 Key Laboratory of Algal Biology, Institute of Hydrobiology, Chinese Academy of Sciences, Wuhan 430072, China; yqfihb@163.com (Q.Y.); liubw@ihb.ac.cn (B.L.); 2 University of Chinese Academy of Sciences, Beijing 100049, China

**Keywords:** *Borodinellopsis*, new species, phylogenetic analysis, 18S rDNA, chloroplast genome, Chlorococcaceae

## Abstract

The genus *Borodinellopsis* is extremely rare and is the subject of limited research and reports. It currently comprises only two species, *Borodinellopsis texensis* and *Borodinellopsis oleifera*, which differ from other globose algae due to their unique centrally radiating chloroplasts. In this study, we describe a new specimen in detail based on morphological data and phylogenetic analysis and identify it as *B. insignis*. *B. insignis* and *B. texensis* exhibit a high degree of similarity, likely due to their shared characteristics of centrally radiating chloroplasts and flagella that are significantly longer than the cell body. A phylogenetic tree constructed based on the 18S rDNA sequence indicates that *B. insignis* and *B. texensis* form a branch that is distinct from other genera, such as *Tetracystis*, *Spongiococcum*, and *Chlorococcum*. Phylogenetic analysis of the ITS sequence, the *rbc*L gene, and the *tuf*A gene reveals that *B. insignis* is significantly different from *B. texensis*, in that it has oil droplets, smaller vegetative cells and zoospores, and distinct habitats. It is also different from *B.oleifera* as it has smaller vegetative cells and zoospores, turns red after cultivation, has longer flagella, and resides in different habitats. The chloroplast genomes of *B. texensis* and *B. insignis* further show significant differences, with the phylogenetic tree constructed based on the analysis of 49 protein-coding genes forming two separate branches. The collinearity of the chloroplast genomes in *B. texensis* and *B. insignis* is poor, with 15 out of the 31 homologous modules displaying inversions and complex rearrangements. Given these differences, we classify this alga as a new species and named it *Borodinellopsis insignis* sp. nov.

## 1. Introduction

Research and reports on *Borodinellopsis* are scarce, and members of this genus are very rare. The two known species of *Borodinellopsis* were both found in coastal soil samples; *B. texensis* was isolated from Mustang Island, Texas (USA) [1], and *B. oleifera* was isolated from the coast of the central Dalmatian island of Lavsa (Croatia, then Yugoslavia) [2]. *Sphaerocystis* [3], *Tetracystis* [4], *Chlorococcum* [5], *Chlorosarcinopsis* [6], *Protococcus* [7], *Hormidium* [8], *Neochloris* [9], and *Spongiosarcinopsis* [10,11], other groups that have been found in soil environments, play an important role in the soil ecosystem, contributing to soil formation and stability [12,13,14,15].

In 1971, Dykstra proposed the genus *Borodinellopsis.* The species of this genus range in shape from spherical to almost spherical, and the characteristic that distinguishes them from other genera in the Chlorococcaceae family is the presence of centrally radiating chloroplasts. Members of this family are widely distributed in soil (e.g., *Pseudopodiococcum*), freshwater (e.g., *Nautococcus* and *Cytomonas*), and marine (e.g., *Valeriella*) environments, exhibiting high cryptic diversity. Many members share the following features: they are capable of producing carotenoids, which give the cells an orange–red color (e.g., *Borodinellopsis* and *Haematococcus*); they produce astaxanthin, a compound known for its anti-cancer effects (e.g., *Chlorococcum* and *Haematococcus*) [16,17,18,19,20]; they exhibit high tolerance to extreme pH levels and high temperatures (e.g., *Chlorococcum*) [20]; and they serve as raw materials for the production of biodiesel (e.g., *Chlorococcum*) [21,22]. In recent years, research on the Chlorococcaceae family has primarily focused on applied and physiological studies, with relatively little attention being given to their taxonomic status [23,24,25,26,27].

The objective of this study was to integrate the phylogenetic results based on 18S rDNA, ITS rRNA sequences, the rbcL gene, and the tufA gene and determine the morphological commonalities and differences among three species of the genus *Borodinellopsis* in order to explore its phylogenetic position. Additionally, a new species, *Borodinellopsis insignis* sp. nov, was proposed, and the structure of its chloroplast genome was discussed.

## 2. Materials and Methods

Algal strain isolation, cultivation, and observation. The FACHB 3529 sample was collected from park wall bricks in Xiaogan, Hubei, China, in January 2020 (31°31′ N, 114°07′ E), and the FACHB 3550 sample was collected from a sandstone surface in Tongliang, Chongqing, China, in March 2014 (29°85′ N, 106°05′ E). Using sterile forceps, we scraped a small amount of each soil sample into a 2 mL centrifuge tube, added an appropriate volume of sterile distilled water, and placed the sample in a vortex mixer for 1–3 min to ensure thorough mixing. Next, we used a pipette to transfer an appropriate volume of the suspension into a sterile BG-11 solid medium containing 1.3% agar and spread the suspension evenly with a glass rod [28]. Next, we placed the culture medium in an incubator at a constant temperature of 25 °C and cultivated it under a light–dark cycle of 12:12 until algal colonies were visible to the naked eye. The next step was to transfer the algal colonies to another sterile culture medium, where we cultivated them again until we obtained a purified single algal colony. Afterwards, we transferred the single algal colony into a 12-well plate containing the BG-11 liquid medium for culture expansion. The algal strains were preserved in the Freshwater Algae Culture Bank (Freshwater Algae Culture Collection at the Institute of Hydrobiology, Chinese Academy of Sciences, China (address: No. 7, Donghu South Road, Wuhan, Hubei)), under accession number FACHB-(3550). Morphological observations of the algal strains were conducted using a Leica DM5000B microscope(Leica Camera AG, Wetzlar, Germany), and photomicrographs were taken using a Leica DFC320 digital camera (Leica Camera AG, Wetzlar, Germany).

DNA extraction, PCR amplification, and sequencing. An appropriate number of algal colonies were placed in a cryovial, and appropriate amounts of buffer solution and bead-beating beads were added. Then, the mixture was disrupted by using a bead beater (model 3110BX; Biospec Products, Buttersville, MI, USA). The resulting solution was transferred into a 2 mL centrifuge tube, and the total DNA was extracted by using an HP Plant DNA Kit (Omega Bio-Tek, GA, USA). PCR amplification was conducted with a reaction volume of 50 μL, which included 6 μL of the template DNA, 1 μL of each primer, and 42 μL of the Master Mix (using the ExTaq enzyme, Takara, Japan). The 18S rDNA sequences were amplified by using the forward primer 18SR and the reverse primer 18SF [29], and the amplification conditions were as follows: incubation at 94 °C for 5 min, followed by a cycling procedure of 94 °C for 50 s, 55 °C for 50 s, and 72 °C for 90 s, and a final extension at 72 °C for 10 min. The ITS sequence was amplified by using primers NS7m and LR1850 [30] under the following amplification conditions: incubation at 94 °C for 5 min, followed by 32 cycles of 94 °C for 1 min, 55 °C for 1 min, and 72 °C for 2 min, and a final extension at 72 °C for 10 min. The PCR products were sent to TSINGKE Biotechnology (Beijing, China) for sequencing, and the sequences were uploaded to GenBank (http://www.ncbi.nlm.nih.gov/) under accession numbers PP544782 and PP574996.

Molecular phylogenetic analyses. Based on the BLAST alignment, the relevant sequences were selected, and a total of 47 18S rDNA sequences, 33 *rbc*L sequences, 20 ITS sequences, and 18 *tuf*A sequences were downloaded from GenBank and then preliminarily aligned by using MAFFT 7.3 [31]. Manual optimization was performed using Seaview (version 5.0) [32]. The pairwise distances were plotted against the model-corrected distances using MEGA (version 11.0) [33] to evaluate the mutational saturation of the alignments in variable positions, and it was found that neither transversion nor transition reached saturation. An analysis was conducted with the maximum likelihood (ML) tool in PhyloSuite (version 1.2.1) [34], and Bayesian inference was performed in MrBayes (version 3.2.2) [35]. The best-fit evolutionary model was selected by using the plugin Modeltest-NG [36]. TN93+G+I, GTR+G+I, GTR+G+I, and GTR+G were found to be the best-fit models for 18S rDNA, ITS, *rbc*L, and *tuf*A, respectively. Detailed information on gene alignment and nucleotide substitution for the maximum likelihood analysis is presented in Table 1. For the maximum likelihood analysis, a tree search was implemented with a heuristic search option that included random sequence addition (10 replicates) and the tree bisection and reconnection branch-swapping algorithm. The statistical reliability was estimated using bootstrap analysis with 1000 replicates of the dataset for the maximum likelihood (ML) analysis. A Bayesian Markov Chain Monte Carlo (MCMC) analysis was conducted, with a total of four Markov chains (three heated chains and one cold chain). Each chain was run for 20 million generations, and tree sampling was performed every 10,000 generations. When the average standard deviation of the split frequencies between two runs was less than 0.01, it was considered that a stationary distribution had been reached. The first 25% of the trees were discarded; then, a consensus tree was constructed by using the remaining samples, and the posterior probabilities were inferred. The editing of the phylogenetic trees was performed by using Figtree 1.4.4 (http://tree.bio.ed.ac.uk/software/figtree/), URL (accessed on 15 December 2021).

Chloroplast genome assembly and annotation. We next performed DNA fragmentation of the detected samples by using the ultrasonic method, followed by the purification and end repair of these samples, and subsequent PCR amplification to generate a sequencing library (Illumina TruSeq v2 DNA Sample Preparation Kit; Catalogue #FC-121-2001). We sequenced the qualified library with Illumina NovaSeq (Illumina Corporation, San Diego, CA, USA) to generate raw data, which were then processed by employing quality control filtering with SOAPnuke software (version 1.3.0) [37] to remove low-quality sequences (reads with N base content exceeding 5%; reads with quality scores of 5 or less, encompassing 50% of the bases; reads contaminated with adapters).

The chloroplast genome was assembled using the SPAdes software 1.1 [38]. The target sequence was determined based on a comparison with known reference chloroplast genomes (*Eudorina elegans*, FACHB 2321, MH161344) [39]. Based on the alignment results of the reads and the collinearity analysis with the chloroplast genome sequences of closely related known species, the relationships among sequences were established. By performing continuous assembly and extension, the complete circular structure of the chloroplast genome was ultimately obtained.

The annotation of the chloroplast genome was performed using the CpGAVAS software [40]. Then, the annotation results were manually corrected using ORF Finder (http://www.ncbi.nlm.nih.gov/orffinder/) URL (accessed on 1 April 2023) and BLAST (http://blast.ncbi.nlm.nih.gov/) URL (accessed on 7 April 2008). The tRNA and rRNA genes were annotated using the tRNAscan-SE 2.0 [41] and RNAmmer [42] online software. To determine the intron boundaries, the gene containing introns was compared with its homologous gene that did not contain introns. Finally, the circular map of the chloroplast genome was obtained using the OrganellarGenomeDRAW (http://ogdraw.mpimp-golm.mpg.de/) URL (accessed on 24 October 2007) online software [43]. The sequences were deposited into GenBank (http://www.ncbi.nlm.nih.gov/) URL (accessed on 8 July 2013) under login number PQ144585.

Chloroplast genome collinearity and phylogenetic analyses. The collinearity analysis was performed with the Mauve version 2.3.1 software [44], and the phylogenetic analysis was carried out by concatenating 49 protein-coding genes from chloroplast genomes, including *atp*A, *atp*B, *atp*E, *atp*F, *atp*H, *atp*I, *ccs*A, *cem*A, *chl*B, *chl*L, *chl*N, *clp*P, *pet*A, *pet*B, *pet*D, *pet*G, *pet*L, *psa*A, *psa*B, *psa*C, *psa*J, *psb*A, *psb*B, *psb*C, *psb*D, *psb*E, *psb*F, *psb*H, *psb*I, *psb*J, *psb*K, *psb*L, *psb*M, *psb*N, *psb*T, *psb*Z, *rbc*L, *rpl*2, *rpl*5, *rpl*14, *rpl*16, *rpl*20, *rpl*23, *rpl*36, *rpo*A, *rpo*C1, *rpo*C2, *rps*2, and *ycf*1. After acquiring the sequence dataset for each gene, sequence alignment was conducted using MAFFT 7.3 [31]. Subsequently, the unaligned regions in the matrix were removed by using Trial 1.2 [45] and were manually adjusted. The dataset was concatenated using SequenceMatrix [46]. The sequence saturation test was carried out using the DAMBE software [47], and the optimal nucleotide substitution model was selected using jModeltest 2.1 [48]. For the phylogenetic analysis, we employed two algorithms: Bayesian inference (BI) and maximum likelihood (ML), where the former was implemented with MrBayes 3.1.2 [49] and the latter with IQ-TREE [50]. The Bayesian analysis was run for 3,000,000 generations, with samples taken every 1000 generations. When the analysis reached stability (variance < 0.01), the operation was terminated. The bootstrap support values and posterior probabilities are displayed on the corresponding branches of the ML and Bayesian trees.

## 3. Results

### 3.1. Borodinellopsis Insignis Q.F. Yan et G.X. Liu sp. nov.

**Figure 1:**
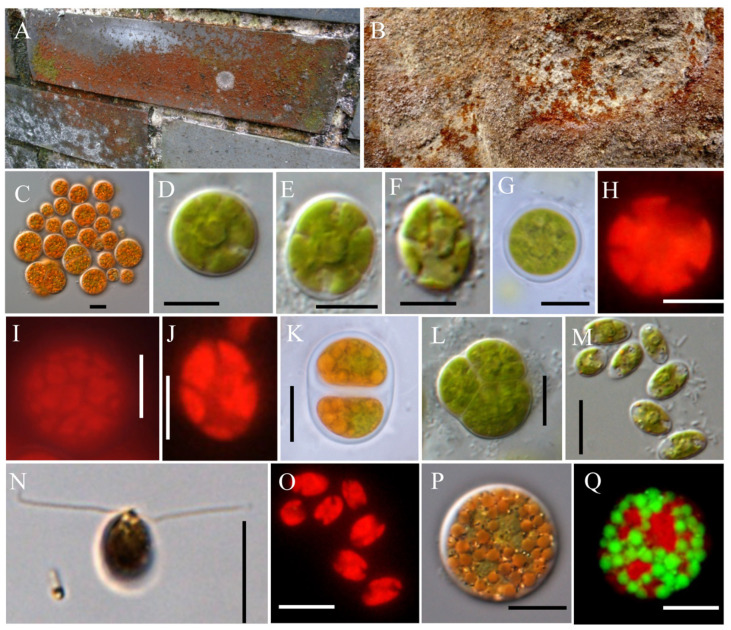
(**A**,**B**) The habitat. (**C**) Vegetative cells. (**D**) Spherical vegetative cells. (**E**) Near-spherical vegetative cells. (**F**) Ellipsoidal vegetative cells. (**G**) Cell wall thickening. (**H**) A single chloroplast. (**I**) Centrally radiating chloroplasts. (**J**) Chloroplasts of ellipsoidal vegetative cells. (**K**) A sporangium containing 2 autospores. (**L**) A sporangium containing 4 autospores. (**M**,**N**) Zoospores. (**O**) Chloroplasts of zoospores. (**P**,**Q**) A large number of orange oil droplets. Scale bar: 10 μm. The cell in (**N**) was fixed and photographed by using Lugol’s solution.

Description: A single vegetative cell, spherical, nearly spherical, or ellipsoidal, with a diameter of 10–24 μm (Figure 1C–F). The cell wall gradually thickened during the culture process (Figure 1G), reaching up to 3 μm, with a centrally radiating chloroplast (Figure 1H–J) and a pyrenoid located at the center of the cell (Figure 1D). In the late stage of cultivation, a large number of oil droplets appeared within the cells, and the cells became orange–red. (Figure 1P,Q). Asexual reproduction occurs through cell division or the formation of autospores and zoospores (Figure 1K–O). The zoospores are 5.5–8 μm in length and 4–5 μm in width, with a cell wall, and the chloroplasts exhibit lobulation (Figure 1M–O). Two flagella are longer than the zoospores (Figure 1N). The sporangium often contains two autospores or four autospores forming a cone shape (Figure 1K,L), and its diameter can reach 28 μm. Sexual reproduction is unknown. 

Etymology: Species names refer to rare and special.

Type locality: Xiaogan (31°31′ N, 114°7′ E), Hubei Province, China; on the surface of park wall bricks.

Iconotype: Figure 1P.

Holotype: QF2020 (HBI), collected by *Q.F. Yan*, March 2014; stored in the Freshwater Algal Herbarium (HBI), Institute of Hydrobiology, Chinese Academy of Sciences, Wuhan, Hubei Province, China.

Distribution: This species grows on the surface of park wall bricks or sandstone surfaces.

Authentic culture: The culture strain (living culture) FACHB-3550 has been deposited and is available at the Freshwater Algae Specimen Station, Institute of Hydrobiology, Chinese Academy of Sciences (http://algae.ihb.ac.cn/). 

Compared with *B. texensis*, *B. insignis* has oil droplets, different habitats, and smaller vegetative cells and zoospores. Unlike *B.oleifera*, *B.insignis* has smaller vegetative cells and zoospores, and lacks sculpted akinetes. The flagella length of *B.oleifera* is about twice the body length, whereas the flagella length of *B.insignis* is twice the body length. Additionally, their habitats are also different: *B. oleifera* was collected from coastal soil, while *B. insignis* was collected from inland soil.

### 3.2. Phylogenetic Analyses

After alignment and trimming, from 52 18S rDNA sequences, we obtained a matrix that included 1771 sites, with 376 (21.2%) being parsimony-informative sites and 490 (27.7%) being variable sites, and from 19 ITS sequences, we obtained a matrix which included 550 sites, with 301 (54.7%) being parsimony-informative sites and 384 (69.8%) being variable sites. 

**Figure 2:**
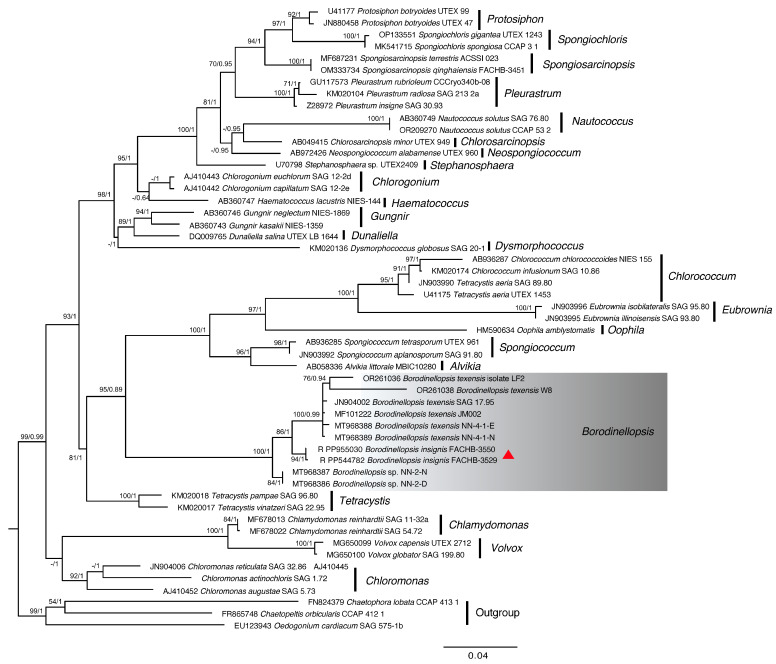
The phylogenetic tree constructed with the Bayesian method based on the 18S rDNA sequences. The Bayesian posterior probabilities and maximum likelihood bootstrap values are shown at the nodes, and the new species from this study is indicated.

**Figure 3:**
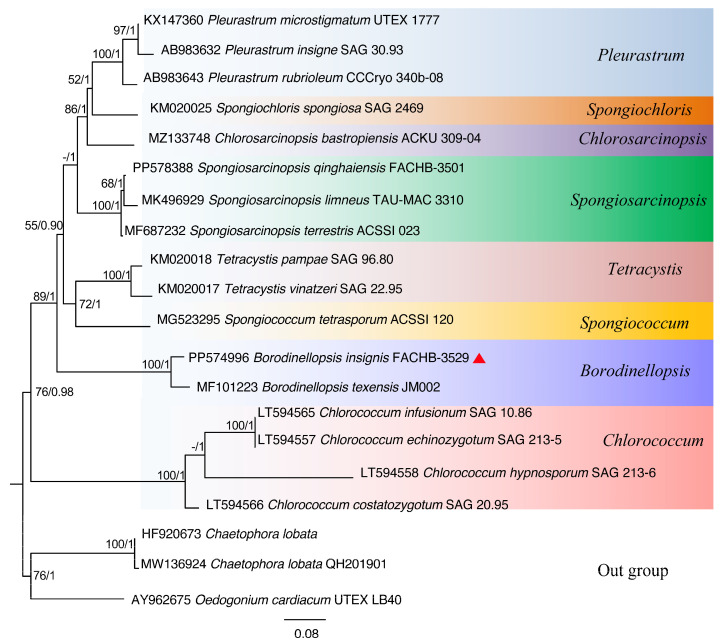
The phylogenetic tree constructed with the Bayesian method based on the ITS sequences. The Bayesian posterior probabilities and maximum likelihood bootstrap values are shown at the nodes, and the new species from this study is indicated.

**Figure 4:**
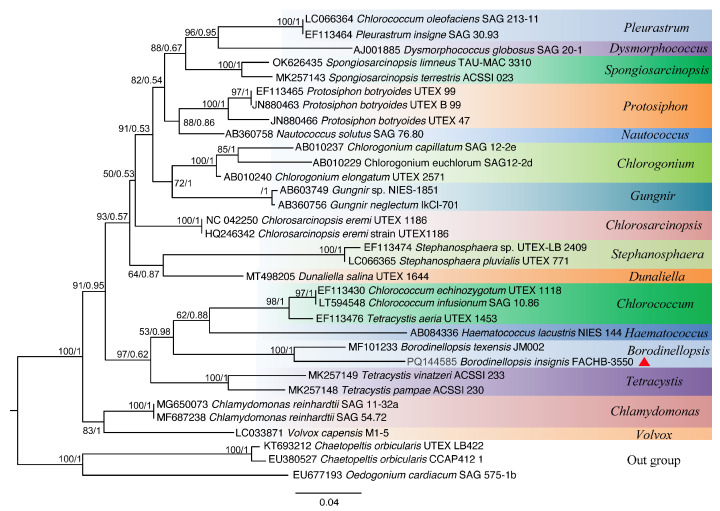
The phylogenetic tree constructed with the Bayesian method based on the *rbc*L sequences. The Bayesian posterior probabilities and maximum likelihood bootstrap values are shown at the nodes, and the new species from this study is indicated.

**Figure 5:**
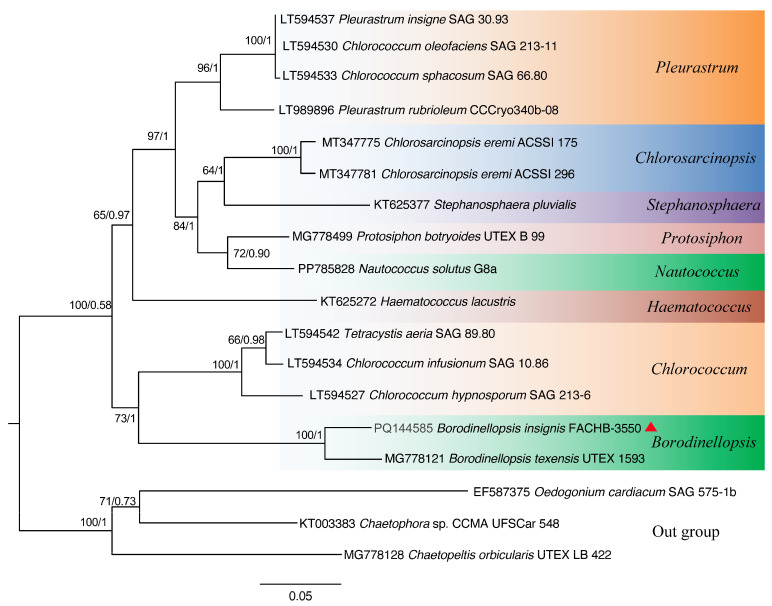
The phylogenetic tree constructed with the Bayesian method based on the *tuf*A sequences. The Bayesian posterior probabilities and maximum likelihood bootstrap values are shown at the nodes, and the new species from this study is indicated.

Based on the nucleotide information shown in Figure 1, a phylogenetic tree was constructed based on the 18S rDNA, ITS, *rbc*L gene, and *tuf*A gene sequences by using the maximum likelihood (ML) and Bayesian inference (BI) methods (Figure 2, Figure 3, Figure 4 and Figure 5). The 18S rDNA phylogenetic tree constructed in this study (Figure 2) is essentially consistent with the topology reported in previous studies [51] and reveals a monophyletic *Borodinellopsis* clade, encompassing the algal strains from the current study; this supports the classification of these species into the genus *Borodinellopsis* (Figure 2). The evolutionary tree constructed based on the ITS sequences depicts the interspecific relationships within the genus *Borodinellopsis*, and the algal strain isolated in the present study forms a distinct branch, clearly separated from *B. texensis* (Figure 3).

### 3.3. Chloroplast Genome Analysis

**Figure 6:**
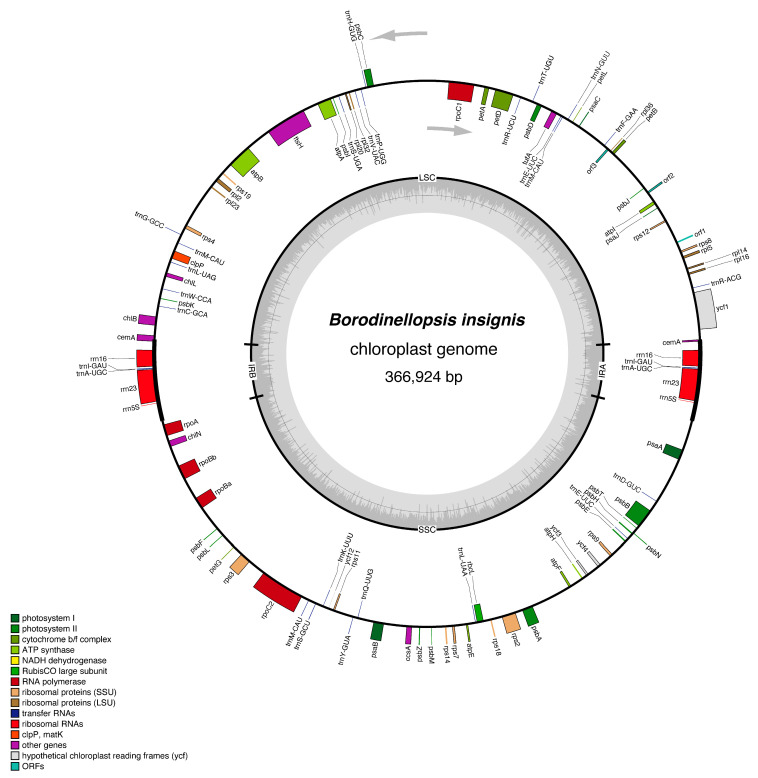
The chloroplast genome map of *Borodinellopsis insignis*, with genes that have different functions indicated by the colors shown in the legend.

In this study, we obtained the complete circular chloroplast genome of this species, representing an addition to the Chlorococcaceae family, and its circular structure map is shown in Figure 6. The chloroplast genome size of *B. insignis* FACHB-3550 is 366,924 bp, with a GC content of 32.54%. The chloroplast genome exhibits a typical tetrad structure, including a large single-copy region of 177,370 bp with a GC content of 31.17%, a small single-copy region of 154,428 bp with a GC content of 32.61%, and two reverse-repeat (IR) regions of 17,563 bp each, with a GC content of 39.17%, and encodes 102 genes, including 67 unique functional genes (one of which is multicopy), three ORFs, 23 tRNA genes (four of which are multicopy), and three rRNA genes (three of which are multicopy); the functional genes include 15 gene families, which are as follows: four genes for subunits of photosystem I (*psa*A, *psa*B, *psa*C, and *psa*J); 15 genes for subunits of photosystem II (*psb*A, *psb*B, *psb*C, *psb*D, *psb*E, *psb*F, *psb*H, *psb*I, *psb*J, *psb*K, *psb*L, *psb*M, *psb*N, *psb*T, and *psb*Z); five genes for subunits of the cytochrome b/f complex (*pet*A, *pet*B, *pet*D, *pet*G, and *pet*L); six genes for subunits of ATP synthase (*atp*A, *atp*B, *atp*E, *atp*F, *atp*H, and *atp*I); one gene for the large subunit of rubisco (*rbc*L); five genes for DNA-dependent RNA polymerase (*rpo*A, *rpo*Ba, *rpo*Bb, *rpo*C1, and *rpo*C2); eight genes for the large subunit of ribosome (*rpl*2, *rpl*5, *rpl*14, *rpl*16, *rpl*20, *rpl*23, *rpl*32, and *rpl*36); 11 genes for the small subunit of ribosome (*rps*2, *rps*3, *rps*4, *rps*7, *rps*8, *rps*9, *rps*11, *rps*12, *rps*14, *rps*18, and *rps*19); one gene for the envelope membrane protein (*cem*A); one gene for the c-type cytochrom synthesis gene (*ccs*A); four genes for open reading frames (*ycf*1, *ycf*3, *ycf*4, and *ycf*12); three genes for subunits of protochlorophyllide reductase (*chl*B, *chl*L, and *chl*N); one gene for the cell division protein FTSH (*fts*H); one gene for protease (*clp*P); and one gene for the translation elongation factor Tu (*tuf*A) (Table 2). It is worth noting that the chloroplast genome of algae is complex and difficult to assemble, and it cannot be guaranteed that the assembled chloroplast genome is circular and complete. In our assembly process, we did our best to ensure the integrity of the genes.

**Figure 7:**
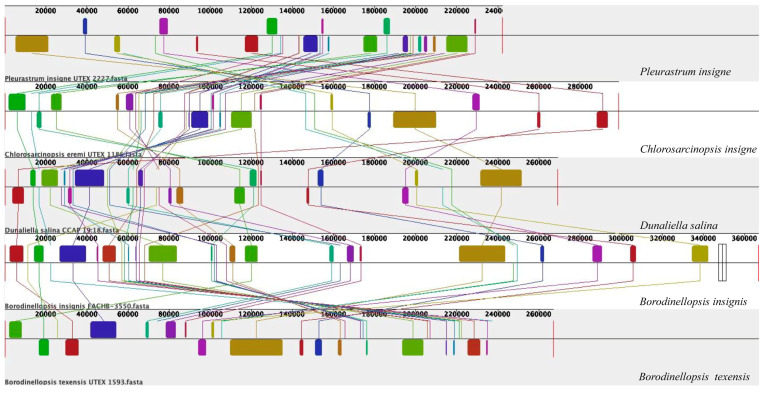
Chloroplast genome collinearity alignment of three species in *Borodinellopsis* and related taxa. *Pleurastrum insigne* (NC042182), *Chlorosarcinopsis insigne* (NC042250), *Dunaliella salina* (GQ250046), *Borodinellopsis insignis* (PQ144585), and *Borodinellopsis texensis* (MG778121).

The collinearity analysis results of the four chloroplast genomes from *Borodinellopsis* and related taxa are shown in Figure 7, arranged in order based on their positions on the constructed phylogenetic tree. The results show that their collinearity is poor, with multiple homologous modules showing rearrangement and inversion, indicating significant differences among the chloroplast genomes of the five species. In order to investigate the degree of collinearity of the chloroplast genomes within the genus *Borodinellopsis*, a collinearity analysis was conducted on two species (*B. insignis* FACHB-3550 and *B*. *texensis* UTEX 1593 = SAG 17.95) within this genus; the results, presented in Figure 7, again indicate poor collinearity, with 9 out of 23 homologous modules being inverted and having undergone complex module rearrangements. 

**Figure 8:**
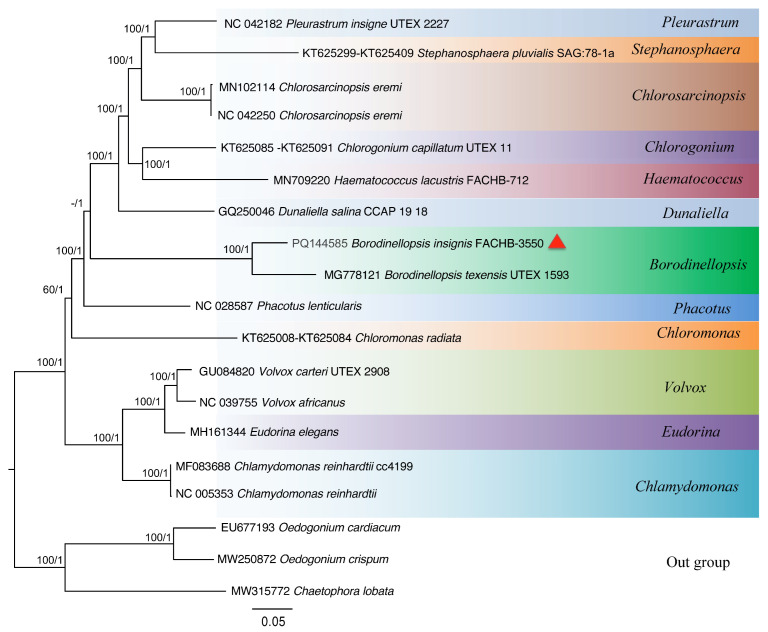
The phylogenetic tree constructed with the Bayesian method based on 49 shared protein-coding genes. Bayesian posterior probabilities and maximum likelihood bootstrap values are shown at the nodes, and the new species from this study is indicated.

In the evolutionary tree constructed based on the 49 common protein-coding gene sequences of the chloroplast genome (Figure 8), the positions of each genus from top to bottom are *Pleurastrum*, *Stephanosphaera*, *Chlorosarcinopsis*, *Chlorogonium*, *Haematococcus*, *Dunaliella*, and *Borodinellopsis*, and each branch has a very high maximum likelihood support value and Bayesian posterior probability. These results are in line with the findings of the phylogenetic studies based on the 18S rDNA sequences.

## 4. Discussion

*Borodinellopsis* belongs to the Chlorococcaceae family. Members of this family are ideal sources of astaxanthin, which has anti-cancer properties (e.g., *Chlorococcum* and *Haematococcus*) [16,17,18,19,20], and also serve as raw materials for biodiesel production (e.g., *Chlorococcum*) [21,22], thereby attracting considerable attention. Therefore, current research on these algae within the Chlorococcaceae family primarily focuses on applied and physiological studies, but there is a relative lack of investigation into their taxonomic status [23,24,25,26,27]. Similar to most species in the Chlorococcaceae family, the existing species within the genus *Borodinellopsis* were also discovered in soil environments. *B. texensis* was isolated from coastal soil samples on Mustang Island, Texas (USA) [1], and *B. oleifera* was isolated from the coast of the central Dalmatian island of Lavsa (Croatia, then Yugoslavia) [2]. Reportedly, members of this family have been found in soil environments, including species such as *Sphaerocystis* [3], *Tetracystis* [4], *Chlorococcum* [5], *Chlorosarcinopsis* [6], *Protococcus* [7], *Hormidium* [8], *Neochloris* [9], and *Spongiosarcinopsis* [10,11]. However, *B.insignis*, in this study, was found on the bricks of a park wall in an inland area, in contrast with *B.oleifera* and *B.texensis,* which were found in coastal soils. This is the first report of the genus *Borodonellopsis* in China. Morphologically, *Borodinellopsis* differs from other genera in the Chlorococcaceae family due to its unique centrally radiating chloroplasts. In this study, we compared *B. insignis* with *B. texensis* and *B. oleifera* within the genus *Borodinellopsis*. Unlike *B. texensis*, *B. insignis* has oil droplets and relatively small vegetative cells and zoospores. Unlike *B. oleifera*, *B. insignis* has smaller vegetative cells and zoospores and lacks sculpted akinetes. The flagella length of *B. oleifera* is about twice the body length, whereas the flagella length of *B. insignis* is twice the body length. Additionally, their habitats are also different: *B. oleifera* was collected from coastal soil, while *B. insignis* was collected from inland soil.

The phylogenetic tree constructed based on the 18S rDNA sequence indicates that *B. insignis* and *B. texensis* form an independent branch of *Borodinellopsis*. This branch, together with *Chlorococcum*, *Eubrownia*, *Oophila*, *Spongiococcum*, *Alvikia*, and *Tetracystis*, constitutes a large clade, indicating that *Borodinellopsis* is an independent genus within the Chlorococcaceae family. This is also confirmed by the constructed phylogenetic tree of 49 common protein-coding genes in chloroplast genomes. Additionally, the phylogenetic tree constructed from the ITS sequences, the *rbc*L gene, and the *tuf*A gene suggests that *B. insignis* and *B. texensis* are two separate species. Due to the lack of molecular sequences for *B. oleifera*, the discussion regarding this species was limited to morphological differences. The analysis of the chloroplast genome of *B. insignis* reveals that similar to other taxonomic groups, such as Scenedesmaceae and Oocystaceae [52], its structure exhibits a typical tetraploid structure. The results of the collinearity analysis indicate that the chloroplast genomes of the genus *Bolodinellopsis* show poor collinearity with those of *Pleurastrom*, Chlorosarcinosis, and Dunaliella. Additionally, there is also poor collinearity between *B. insignis* and *B. texensis*. Complex genome inversions and rearrangements also occur in other algae within the Volvocales, for example, between *Eudorina elegans* and *Eudorina cylindrica* [39]. The inversions and rearrangements of multiple homologous modules among different genera and species indicate significant differences in the chloroplast genome. The phylogenetic analysis based on the 49 common genes in the chloroplast genomes supports the findings of independence and correlation among *Borodinellopsis* genera and species from the phylogenetic analyses of 18S rDNA, ITS sequences, the *rbc*L gene, and the *tuf*A gene.

In summary, *B. insignis* was identified as a new species within the genus *Borodinellopsis*. This study’s results enrich the diversity of the genus *Borodinellopsis* and simultaneously introduce a new chloroplast genome, which provides a reference and data support for further research into the relationships and boundaries among genera within the Chlorococcaceae family.

## Figures and Tables

**Table 1:** Detailed nucleotide information for constructing a phylogenetic tree based on 18S rDNA, ITS sequences, the *rbc*L gene, and the *tuf*A gene.

Dataset	18S rDNA	ITS	*rbc*L	*tuf*A
Alignment length	1771	436	997	676
Number of sequences	52	20	33	18
Parsimony-informative sites	376	217	328	245
Invariant sites	1281	180	592	349
Best-fit model	TN93+G+I	GTR+G+I	GTR+G+I	GTR+G
Base frequency% (T/C/A/G)	26/21/25/28	23/25/27/25	31/19/28/22	31/14/34/21
Saturation test (*Iss/Iss.c*)	0.121 < 0.837	0.572 < 0.700	0.371 < 0.753	0.213 < 0.739

**Table 2:** Genes encoded by *Borodinellopsis insignis* chloroplast genome.

Gene Product	Gene
Subunits of photosystem I	*psa*A, -B, -C, -J
Subunits of photosystem II	*psb*A, -B, -C, -D, -E, -F, -H, -I, -J, -K, -L, -M, -N, -T, -Z
Subunits of cytochrome b/f complex	*pet*A, -B, -D, -G, -L
Subunits of ATP synthase	*atp*A, -B, -E, -F, -H, -I
Large subunit of rubisco	*rbc*L
Small subunit of ribosome	*rps*2, -3, -4, -7, -8, -9, -11, -12, -14, -18, -19
Large subunit of ribosome	*rpl*2, -5, -14, -16, -20, -23, -32, -36
DNA-dependent RNA polymerase	*rpo*A, -Ba, -Bb, -C1, -C2
rRNA genes	rrn5S(×2), -16S(×2), -23S(×2)
Envelope membrane protein	*cem*A(×2)
Protease	*clp*P
c-type cytochrome synthesis gene	*ccs*A
Genes of unknown functions Open Reading	*ycf*1, -3, -4, -12
Subunits of protochlorophyllide reductase	*chl*B, -L, -N
Cell division protein FTSH	*fts*H
Translation elongation factor Tu	*tuf*A
tRNA genes	trnA-UGC(×2), -C-GCA, -D-GUC, -E-UUC(×2), -F-GAA, -G-GCC, -H-GUG, -I-GAU(×2), -K-UUU, -L-UAA, -L-UAG, -M-CAU(×3), -N-GUU, -P-UGG, -Q-UUG, -R-ACG, -R-UCU, -S-GCU, -S-UGA, -T-UGU, -V-UAC, -W-CCA, -Y-GUA

## Data Availability

Data are contained within the article.
